# Epicardial-to-Endocardial Activation Gradients and Conduction Block During Atrial Fibrillation in the Human Left Atrial Posterior Wall

**DOI:** 10.1161/CIRCEP.125.014151

**Published:** 2026-04-03

**Authors:** Christopher X. Wong, Xiang Wen Lee, Nitish Badhwar, Chikezie K. Alvarez, Anson M. Lee, Christopher E. Woods, Thomas A. Dewland, Edward P. Gerstenfeld, Ramin E. Beygui, Randall J. Lee

**Affiliations:** 1 Department of Cardiology, University of Adelaide and Royal Adelaide Hospital, Australia (C.X.W.).; 2 Division of Electrophysiology, Department of Cardiology (X.W.L., T.A.D., E.P.G., R.J.L.) and Division of Cardiothoracic Surgery, University of California San Francisco.; 3 Division of Electrophysiology, Cardiothoracic Surgery, Cardiovascular Research Center, Stanford University, Palo Alto, CA (N.B., C.K.A.).; 4 Division of Cardiothoracic Surgery, University of California, San Francisco (R.E.B).; 5 Cardiothoracic Surgery, Cardiovascular Research Center, Stanford University, Palo Alto, CA (A.M.L.).; 6 Atrial Fibrillation and Complex Arrhythmia Center, Sutter California Pacific Medical Center, San Francisco (C.E.W.).

**Keywords:** atrial appendage, atrial fibrillation, catheters, epicardial mapping, pulmonary veins

## Abstract

**BACKGROUND:**

Although emerging evidence supports 3-dimensional myocardial activation during atrial fibrillation (AF), human studies remain limited. We thus characterized the endocardial and epicardial left atrial posterior wall (LAPW) in humans to assess the prevalence of asynchronous endocardial-epicardial LAPW conduction during AF.

**METHODS:**

Patients with symptomatic nonparoxysmal AF who had unsuccessful antiarrhythmic or catheter ablation therapy referred for hybrid epicardial-endocardial AF ablation and left atrial appendage ligation underwent high-density mapping of LAPW with Grid catheters, including simultaneous endocardial-epicardial mapping.

**RESULTS:**

Twenty-seven patients (19 men, median 69 years, 55% long-standing persistent AF) were included. There was significantly greater epicardial compared with endocardial LAPW bipolar voltages during AF. In areas of low endocardial bipolar voltage, normal endocardial unipolar voltage corresponded to normal epicardial bipolar voltage. Asynchronous endocardial-epicardial LAPW AF activation during simultaneous endocardial-epicardial mapping was universal. Furthermore, more rapid epicardial compared with endocardial LAPW AF activity was observed during simultaneous endocardial-epicardial mapping in AF. Conduction block between the endocardial and epicardial LAPW surfaces was also common during organized AF, with instances of isolated or multiple blocked beats, Wenckebach conduction, and sustained endocardial LAPW entrance block with ongoing epicardial AF observed. Epicardial-to-endocardial entrance block was also infrequently observed during sinus rhythm. At 12-month follow-up, freedom from atrial arrhythmias was 68%.

**CONCLUSIONS:**

Endocardial-epicardial LAPW asynchrony may be observed during human persistent AF and is characterized by: (1) greater epicardial compared with endocardial bipolar voltages, (2) more frequent epicardial-to-endocardial activation gradients during AF, and (3) conduction block commonly seen between the epicardial and endocardial surfaces during AF. Although the study was predominantly descriptive in nature, the observations suggest a dynamic 3-dimensional arrhythmogenicity of the LAPW and the potential importance of the epicardial layer, with implications for ablation therapies. Future prospective studies are required to determine the significance of these findings to clinical ablation outcomes.

**What is Known?**

- The mechanisms underpinning persistent atrial fibrillation remain incompletely defined, limiting the strategies and long-term outcomes of ablation in this population.
- Observations from experimental models, and superior results from hybrid ablation strategies, suggest that both the endocardial and epicardial layers of the left atrial posterior wall may be important in atrial arrhythmogenesis. However, limited mapping data from both the endocardial and epicardial layers in humans is available.

**WHAT THIS STUDY ADDS**

- Patients undergoing hybrid ablation and left atrial appendage ligation underwent high-density endocardial and epicardial mapping of the left atrial posterior wall.
- Multiple phenomena consistent with endocardial-epicardial asynchrony were seen during persistent atrial fibrillation, including voltage discrepancies, activation gradients, and conduction block.
- These observations suggest a dynamic 3-dimensional arrhythmogenicity of the left atrial posterior wall with implications for ablation strategies.

Atrial fibrillation (AF) is the most common cardiac arrhythmia and is associated with stroke, heart failure, dementia, and death.^1^ Endocardial catheter ablation is effective in paroxysmal AF and has become a first-line therapy.^2,3^ However, endocardial catheter ablation has produced suboptimal results in nonparoxysmal AF, especially in patients with advanced nonparoxysmal AF or associated atrial myopathy.^4–6^

An enlarged left atrium is associated with an increased prevalence of AF^7–11^ and is a recognized risk for AF recurrence after endocardial catheter ablation.^12–17^ AF-induced left atrial posterior wall (LAPW) remodeling disrupts the atrial myocyte syncytium with an increase in interstitial fibrosis, creating anatomic layers of heterogeneous activation.^18–20^ Asynchronous endocardial-epicardial LAPW activation has been hypothesized to be an important contributor to AF maintenance by increasing the left atrial mass and areas of slow conduction, favoring reentry. Recurrence of AF in advanced nonparoxysmal AF after endocardial catheter ablation is thought to be due to the lack of durable transmural lesions, allowing epicardial-endocardial asynchronous activation to persist, thus permitting areas of reentry to occur.^21^

In this study, we performed endocardial and epicardial mapping during a hybrid epicardial-endocardial ablation strategy to assess the prevalence of asynchronous conduction and characterize conduction between the epicardial and endocardial LAPW during AF.

## Methods

### Study Population

The data that support the findings of this study are available from the corresponding author on reasonable request. Patients with persistent or long-standing persistent AF who had unsuccessful antiarrhythmic or catheter ablation therapy, undergoing hybrid epicardial ablation and left atrial appendage (LAA) exclusion, were prospectively recruited from 2 centers. Persistent AF was defined as AF that is continuously sustained beyond 7 days. Early persistent AF was defined as AF lasting <3 months, and late persistent AF lasting between 3 and 12 months. Long-standing persistent AF is defined as continuous AF ≥1 year in duration in whom rhythm control management is being pursued.^22^ Key exclusion criteria included New York Heart Association class IV heart failure, body mass index >42, prior open heart surgery or intervention in the pericardial space, and documented thromboembolic event, myocardial infarction, or unstable angina within 3 months of enrollment. All patients provided written informed consent to the procedure and the study protocol. The study was approved by the University of California, San Francisco, and Stanford University institutional review boards.

### Hybrid Mapping/Ablation and LAA Exclusion Procedure

All procedures were performed under general anesthesia. Patients had a preprocedure computerized tomography angiogram and a transthoracic echocardiogram. Anticoagulation was held 2 days before the procedure with subsequent low molecular weight heparin bridging. Intraprocedural transesophageal echocardiography confirmed the absence of LAA or other intracardiac thrombi.

**Figure 1.**
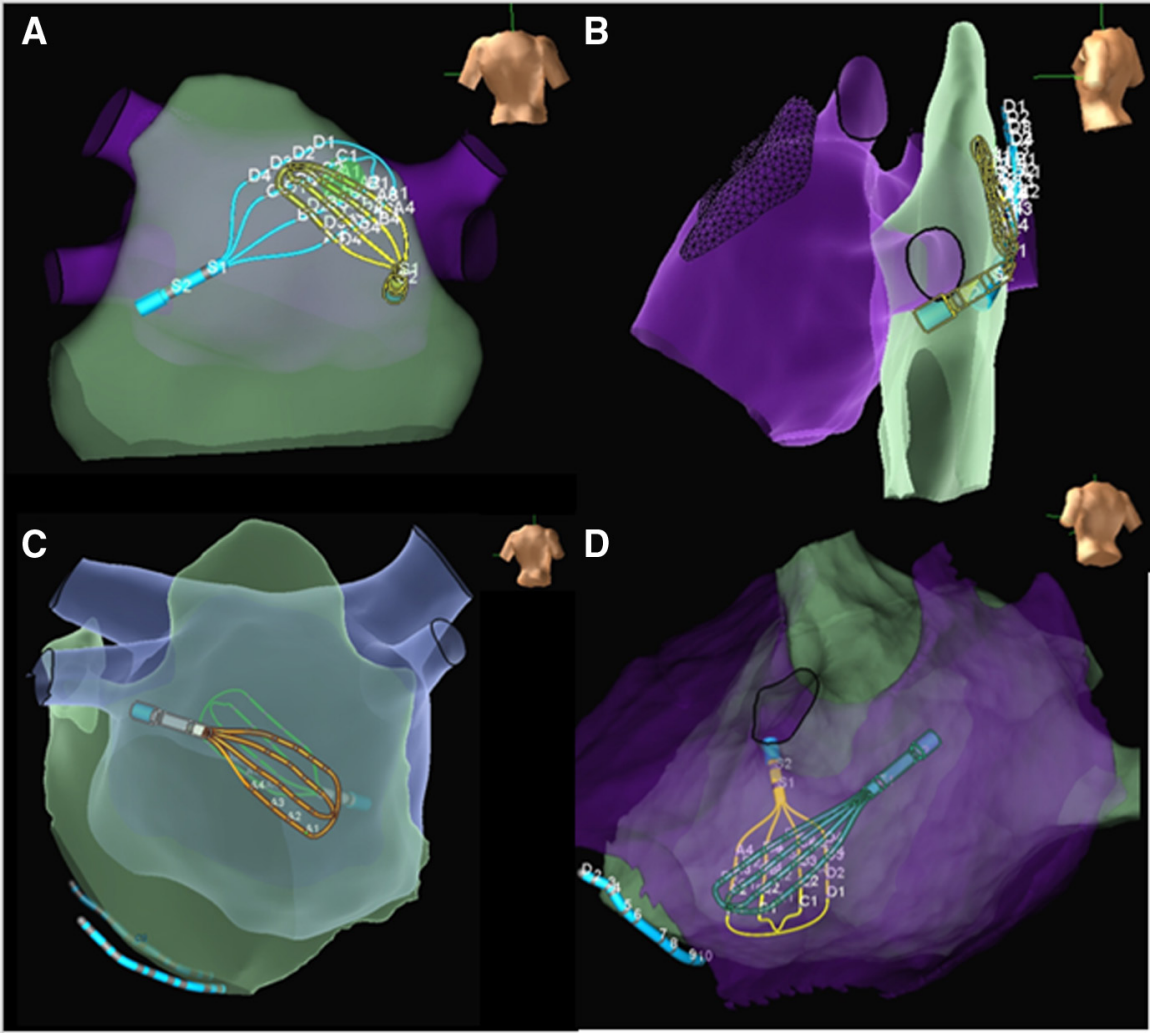
**Three-dimensional maps demonstrating standardized positions for simultaneous endocardial and epicardial mapping.** Representative 3-dimensional maps demonstrating standardized positions for simultaneous endocardial and epicardial mapping: mid-posterior wall (**A** and **B**), mid-inferior left atrium (**C**), inferolateral left atrium (**D**). Overlapping multipolar catheters (Advisor HD Grid, Abbott) are shown on the endocardial (purple) and epicardial (green) left atrial surfaces.

**Figure 2.**
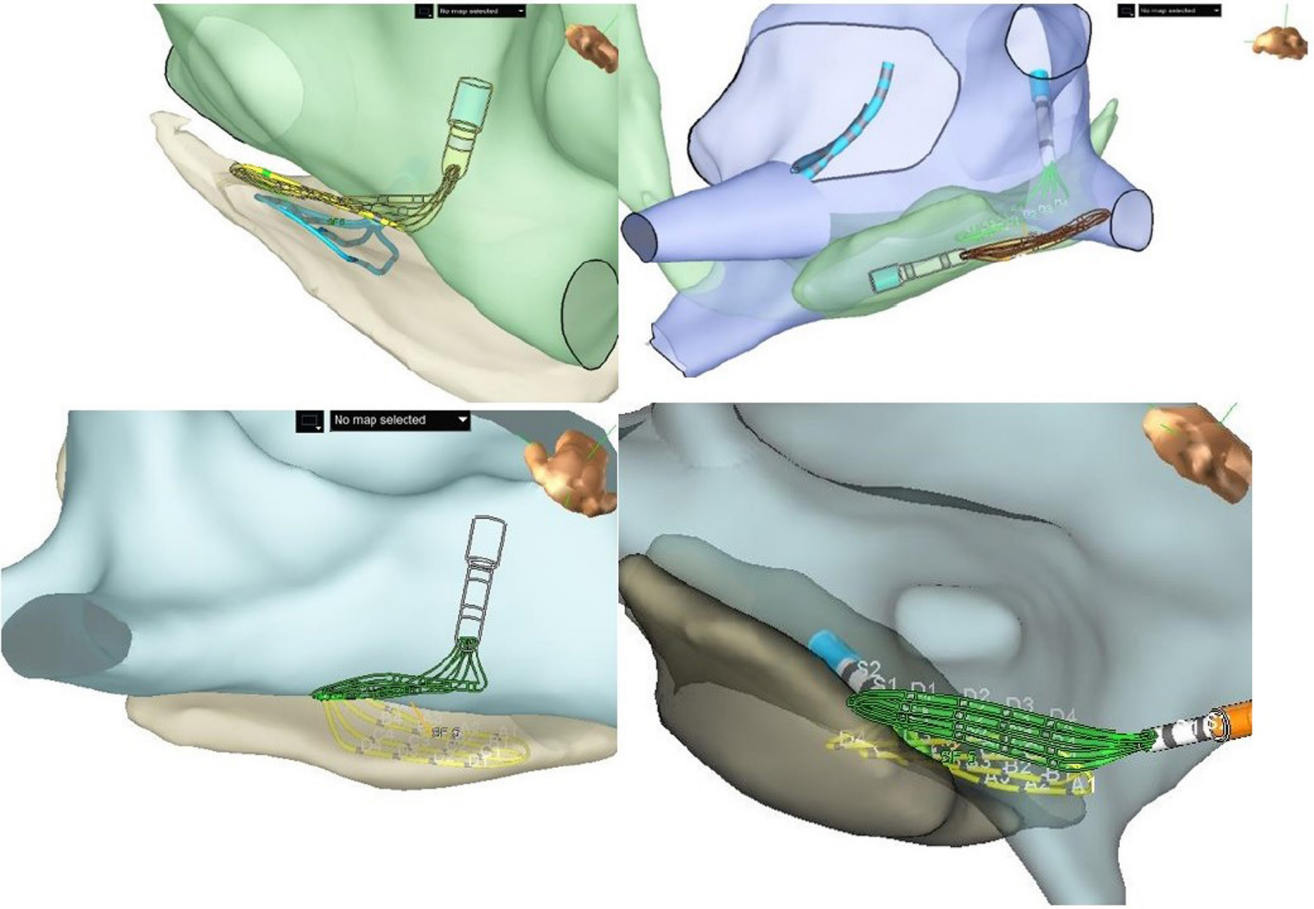
**Catheter proximity for simultaneous endocardial and epicardial mapping.** Representative 3-dimensional maps demonstrating the proximity of multipolar catheters (Advisor HD Grid, Abbott) during simultaneous endocardial and epicardial mapping. Distances between catheters are measured and vary between 4 and 5 mm.

Subxiphoid epicardial access was obtained percutaneously or via a surgical window. A decapolar catheter was positioned in the coronary sinus. Transseptal puncture was performed after heparinization (target activated clotting time 300–350 seconds). Epicardial LAA ligation was first performed as previously described.^23^ After the completion of LAA ligation, endocardial electroanatomical mapping was performed with the Abbott Ensite NavX Precision system (Abbott Laboratories, Abbott Park, IL). An Abbott Advisor HD Grid mapping catheter was subsequently advanced to the endocardial and epicardial LAPW surfaces. In a subset of individuals, a second Advisor HD Grid mapping catheter was used for simultaneous endocardial and epicardial LAPW mapping. A minimum of 3 LAPW locations (mid-posterior wall, mid-inferior left atrium, inferolateral left atrium) were systematically sampled with catheters manipulated to optimize alignment and overlap of recording splines (Figure 1). Catheter positioning consisted of catheter deflection >60 degrees consistent with adequate contact, and minimal distance (≤5 mm) between overlapping catheters was confirmed with both fluoroscopy and 3-dimensional mapping (Figure 2). Simultaneous activation during sinus rhythm, AF, or organized atrial flutter at each location for 60 seconds was documented with bipolar recordings (30–500 Hz). The frequency of the mapped endocardial and epicardial electrograms was quantitatively estimated using the automated fractionation algorithm within the NavX Precision mapping system with parameters of 10 ms for width, 30 ms for refractory period, and 8 deflections for threshold.^24^ At each location, bipolar voltage data from 32 orthogonal points were also collected for quantitative analysis. Location-specific endocardial and epicardial voltage and frequency data were quantitatively compared, excluding data points that were >5 mm from the opposing surface. The initial 6 patients had simultaneous endocardial-epicardial mapping, while the remaining patients had sequential endocardial then epicardial LAPW mapping with the Grid catheter. Unipolar voltage maps of the endocardial LAPW were also obtained from patients with simultaneous endocardial-epicardial mapping.

**Figure 3.**
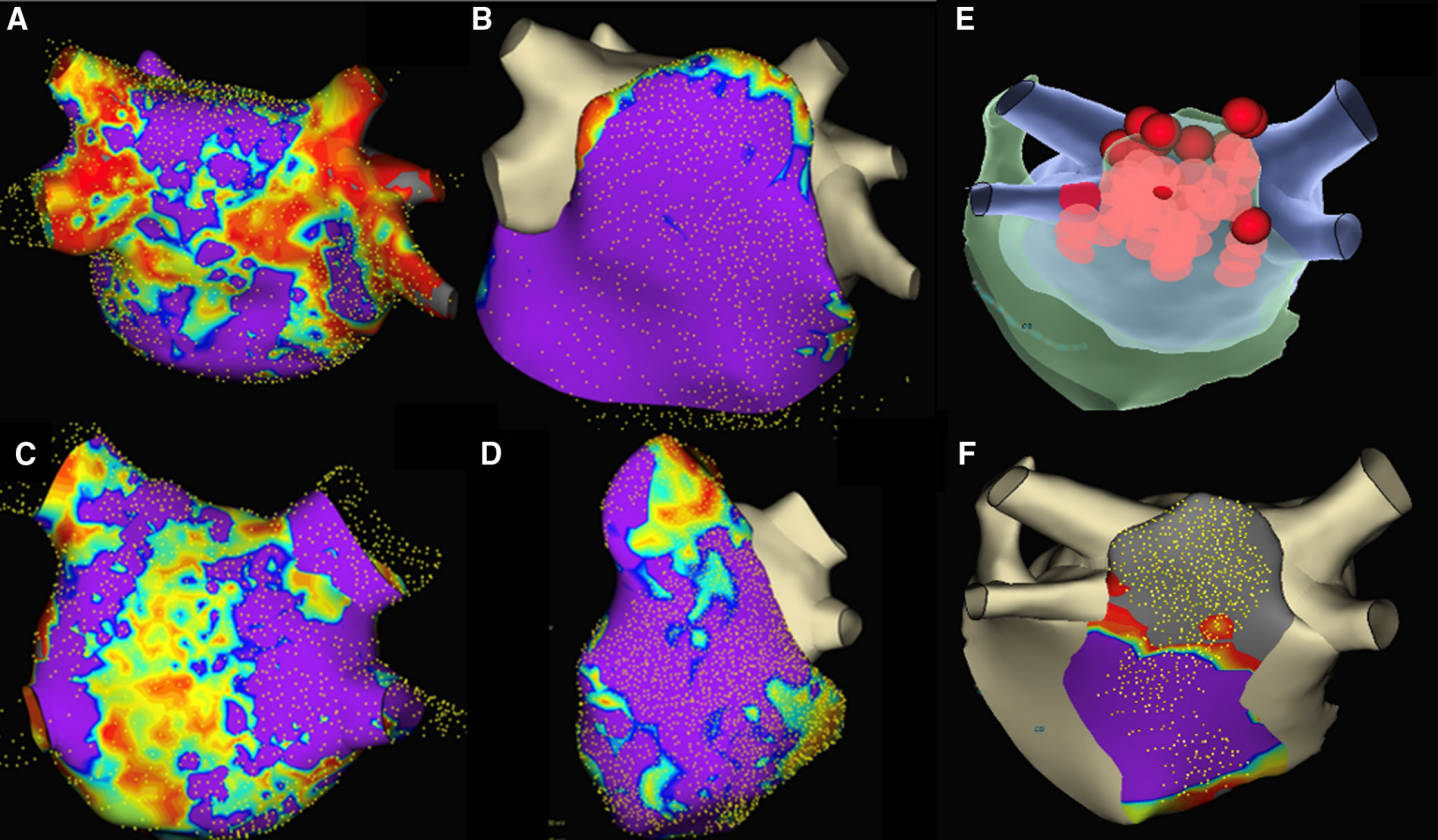
**Representative examples of epicardial-endocardial bipolar voltage discrepancy during atrial fibrillation from 2 de novo patients with simultaneous epicardial and endocardial mapping, and an example of epicardial ablation. A** and **C**, Endocardial bipolar voltage maps, and **B** and **D** demonstrate epicardial bipolar voltage maps. **E**, Typical lesion set for epicardial ablation. **F**, Postepicardial ablation map. Voltage scale from 0.05 mV (red) to 0.5 mV (purple).

Once initial mapping was completed, a subxiphoid surgical window was created if not already performed and epicardial LAPW ablation was undertaken with an EPi-Sense ablation device (AtriCure Inc, OH).^25^ Epicardial ablation was guided by 3-dimensional geometry and direct camera visualization, supplanted with repeat epicardial mapping to assess for appropriate voltage reduction (Figure 3E and 3F). If the patient remained in an atrial arrhythmia after epicardial ablation, external cardioversion to sinus rhythm was undertaken. After hospital discharge, patients returned for a staged endocardial ablation procedure 6 to 12 weeks later. The final hybrid lesion set included pulmonary vein isolation (PVI), LAPW isolation, cavo-tricuspid isthmus line, and LAA ligation.

### Postprocedural Care and Follow-Up

Oral anticoagulation therapy was restarted within 4 hours of completing the hybrid epicardial ablation and LAA ligation procedure and continued uninterrupted through the staged endocardial catheter ablation procedure. Colchicine (0.6 mg twice a day) and NSAIDS were initiated and continued for at least 3 weeks. Adverse events were collected at 7 days and 30 days. Long-term embolic events and mortality status were also collected. After a blanking period of 3 months, all antiarrhythmic drugs were discontinued. To assess for atrial arrhythmias, 7-to-14-day continuous monitoring was performed at 6 months and 1 year, or earlier if the patient had symptoms. Anticoagulation therapy was continued unless there was a contraindication to long-term therapy.

### Statistical Analysis

Continuous variables were presented as mean (±SD) or median (interquartile range), as appropriate to distribution, and categorical variables as number and percentage. Quantitative analysis of bipolar voltage and frequency data from endocardial and epicardial electrograms at 3 locations (mid-posterior wall, mid-inferior left atrium, inferolateral left atrium) during AF and sinus rhythm was undertaken. One-way ANOVA was used for the 3 location comparison (overall difference), and paired *t* tests used for paired location comparisons. Adjustment for multiple comparisons was not undertaken in view of the exploratory nature of the work. Analyses were conducted using Stata (version 18) and statistical significance set at *P*<0.05.

## Results

### Patient Characteristics

Between 2021 and 2024, a total of 27 patients (19 men, median 69 years [interquartile range, 64–75]) with nonparoxysmal AF refractory to either antiarrhythmic drugs or previous catheter ablation who underwent a subxiphoid hybrid epicardial ablation, LAA ligation, and endocardial-epicardial mapping were included (Table). A majority of patients had long-standing persistent AF (55%). A de novo hybrid procedure was performed in 18 of 27 patients. Patients with prior ablation were not excluded if the posterior wall had previously undergone ablation. However, none of the patients included actually had prior posterior wall isolation undertaken. Median transthoracic echocardiography left atrial volume index was 40 mL/m^2^ (interquartile range, 34–48), whereas median computed tomography or magnetic resonance imaging left atrial volume was 190 mL (152–227). Median left ventricular ejection fraction was 56% (interquartile range, 40–60). Two patients had a history of LAA thrombus.

**Table.**
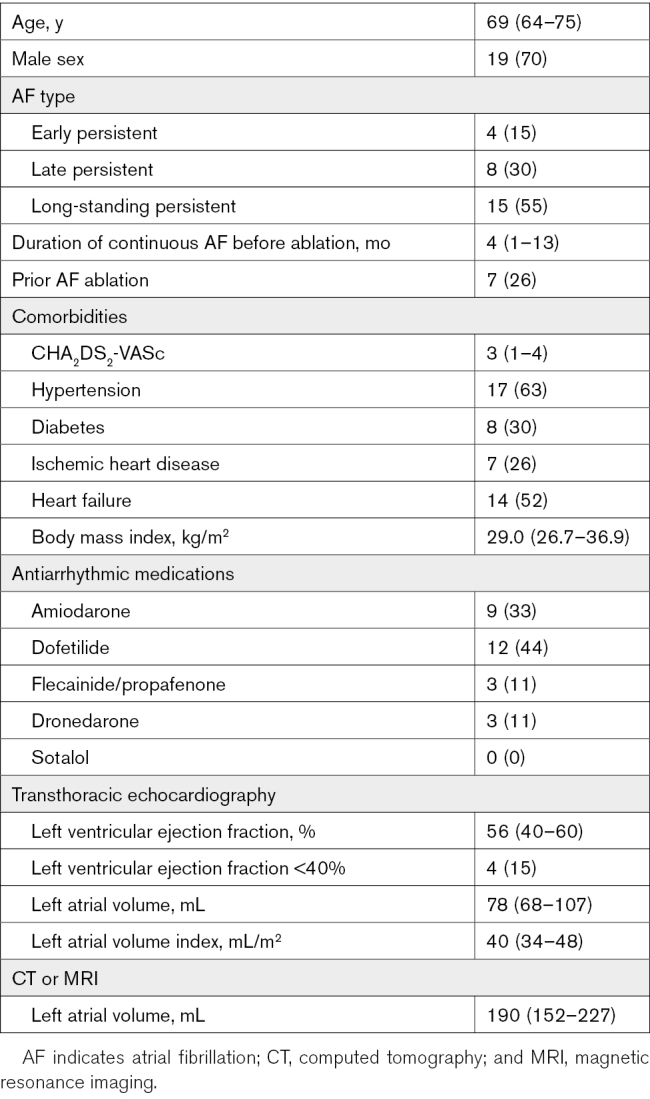
Baseline Characteristics of the Study Population

### Endocardial and Epicardial Bipolar Voltage Mapping

**Figure 4.**
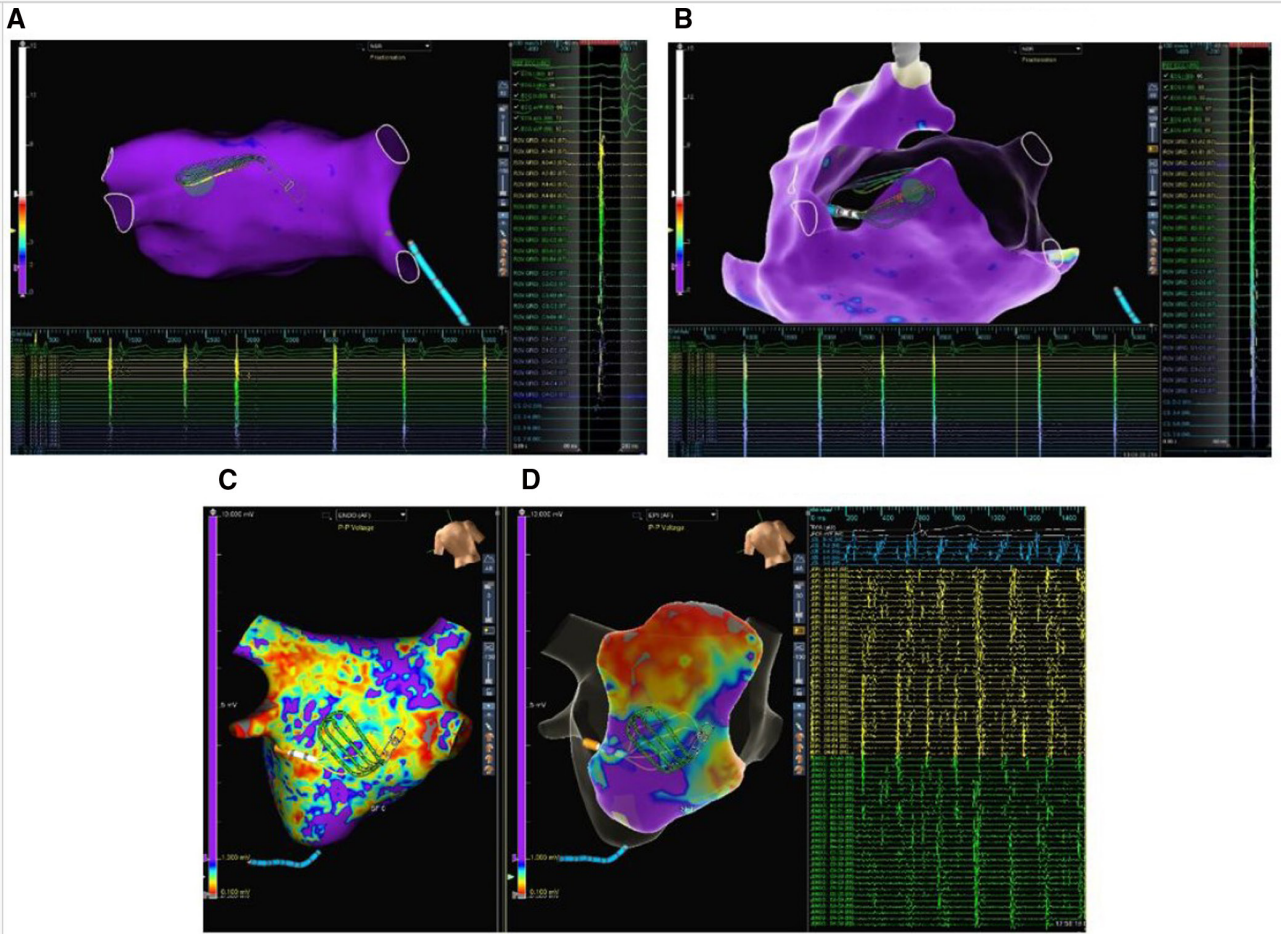
**Simultaneous bipolar epicardial and endocardial voltage mapping. A**, Sinus rhythm (SR) endocardial (Endo) bipolar voltage**. B**, SR epicardial (Epi) bipolar voltage. **C**, Atrial fibrillation (AF) Endo bipolar voltage. **D**, AF Epi bipolar voltage. Bipolar voltage maps during SR and AF from a patient with simultaneous Epi and Endo mapping. **A** and **B** demonstrate Endo and Epi bipolar voltage maps, respectively, during SR, which show relatively healthy and comparable bipolar voltage. **C** and **D** demonstrate Endo and Epi bipolar voltage maps, respectively, during AF, which show lower voltage compared with sinus rhythm and slightly greater regions of preserved Epi bipolar voltage compared with the endocardium.

Twenty-six patients underwent high-density mapping of the endocardial and epicardial LAPW surfaces, with 1 patient having only mapping of the endocardial LAPW surface. Of these 26 patients, 16 were mapped during AF and 10 while in sinus rhythm. Cardioversion to sinus rhythm was attempted in patients presenting in AF, but this was limited by the persistent AF nature of these individuals, and thus, mapping in both AF and sinus rhythm was only achieved in 1 patient. Six patients underwent simultaneous endocardial-epicardial mapping with 2 separate catheters, with the remaining 20 patients having sequential mapping of the LAPW surfaces. The median distance of simultaneous endocardial-epicardial mapping catheters was 5 mm (interquartile range, 4.75–5.75). Among the 16 patients mapped during AF, all demonstrated greater epicardial compared with endocardial bipolar voltage (Figure 3A through 3D). Quantitative analysis during simultaneous endocardial-epicardial LAPW mapping in AF demonstrated a significant difference in bipolar voltages, with epicardial greater than endocardial bipolar voltage at the mid-posterior wall (0.73±0.64 versus 0.23±0.13 mV; *P*=0.005), mid-inferior left atrium (1.23±1.12 versus 0.36±0.53; *P*=0.01), and inferolateral left atrium (1.23±1.13 versus 0.37±0.52; *P*=0.02). Among individuals in whom both sufficient endocardial and epicardial LAPW mapping was able to be undertaken during sinus rhythm, epicardial and endocardial bipolar voltages were not significantly different at the mid-posterior wall (3.43±1.91 versus 1.30±1.26 mV; *P*=0.21), mid-inferior left atrium (4.20±2.20 versus 2.61±2.75; *P*=0.66), and inferolateral left atrium (4.09±2.29 versus 1.40±1.22; *P*=0.17). Epicardial and endocardial voltages were greater during sinus rhythm compared with during AF (*P*<0.05 for all sites; Figure 4).

### Endocardial and Epicardial Unipolar Voltage Mapping

**Figure 5.**
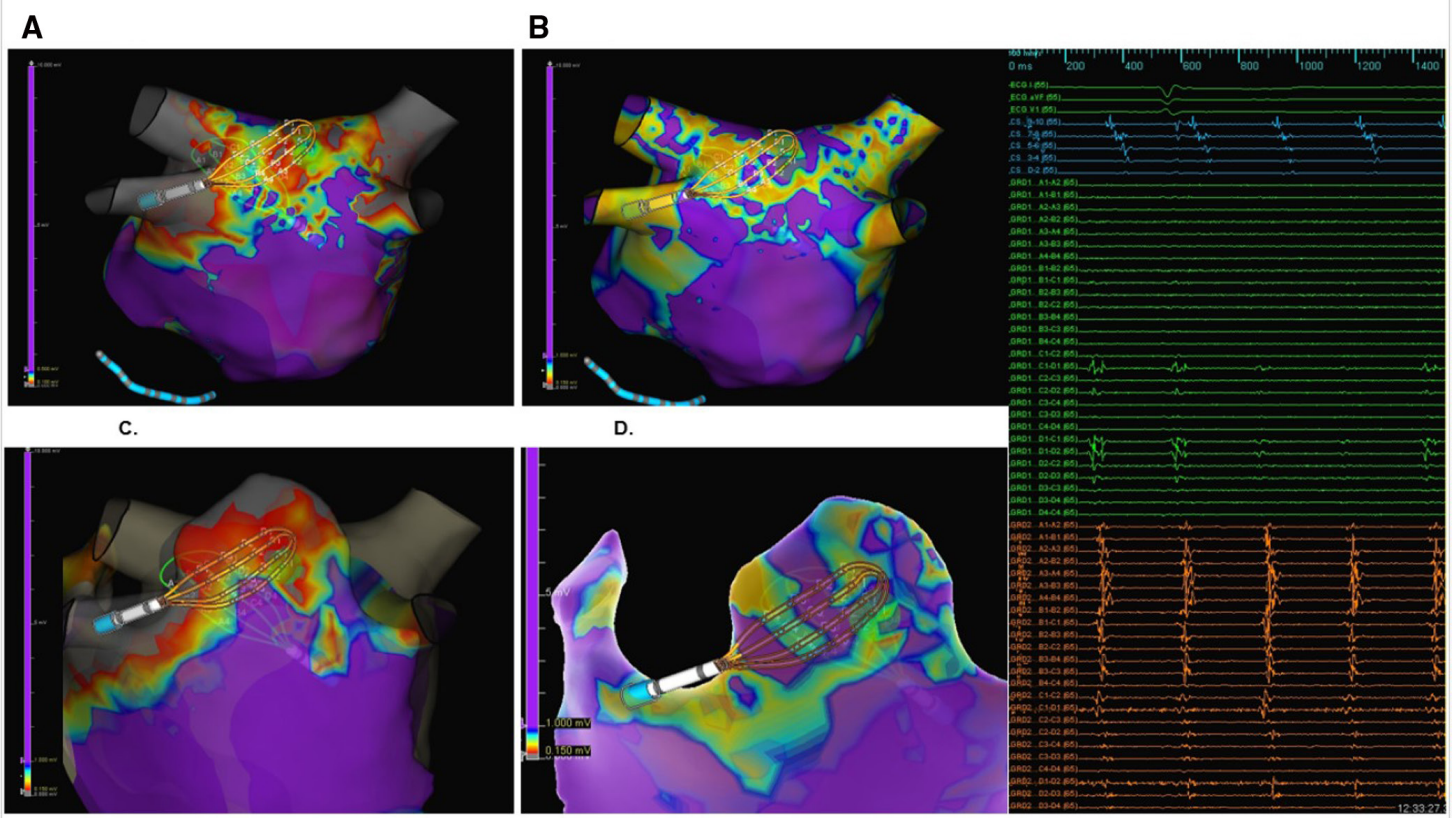
**Simultaneous epicardial and endocardial bipolar and unipolar voltage mapping. A**. Atrial fibrillation (AF) endocardial (Endo) bipolar voltage. **B**, AF Endo unipolar voltage. **C**, AF epicardial (Epi) bipolar voltage. **D**, AF Epi unipolar voltage. Representative example of bipolar and unipolar Endo and Epi maps derived during simultaneous Epi-Endo mapping. Endo and Epi bipolar voltage maps (**A** and **C**), as well as Endo and Epi unipolar voltage maps (**B** and **D**), are shown. The unipolar Endo map (**B**) shows some areas where there is low Endo bipolar voltage (**A**) but greater Endo unipolar voltage that corresponds to greater Epi bipolar and unipolar voltage (**C** and **D**). The Endo and Epi HD grid catheter positioning corresponds to simultaneous Endo and Epi recordings. Simultaneous Endo recordings (green electrograms) demonstrate areas of low to no electrograms as compared with the Epi electrograms (orange recordings). Bipolar voltage scale from 0.05 mV (red) to 0.5 mV (purple). Unipolar voltage scale from 0.15 mV (red) to 1.0 V (purple).

Unipolar voltage maps were obtained during simultaneous endocardial and epicardial LAPW mapping using 2 separate Grid catheters in 6 patients during AF. Areas of normal endocardial unipolar voltage were frequently observed in areas of low endocardial bipolar voltage with no endocardial electrical potentials (Figure 5). Simultaneous epicardial mapping in areas of normal endocardial unipolar voltage commonly demonstrated normal epicardial bipolar voltage and electrical potentials. In contrast, areas of normal endocardial bipolar voltage generally had comparably normal endocardial unipolar voltages and electrical potentials corresponding to similarly normal epicardial bipolar voltage and electrical activity.

### Endocardial and Epicardial Posterior Left Atrial Activation Frequency

**Figure 6.**
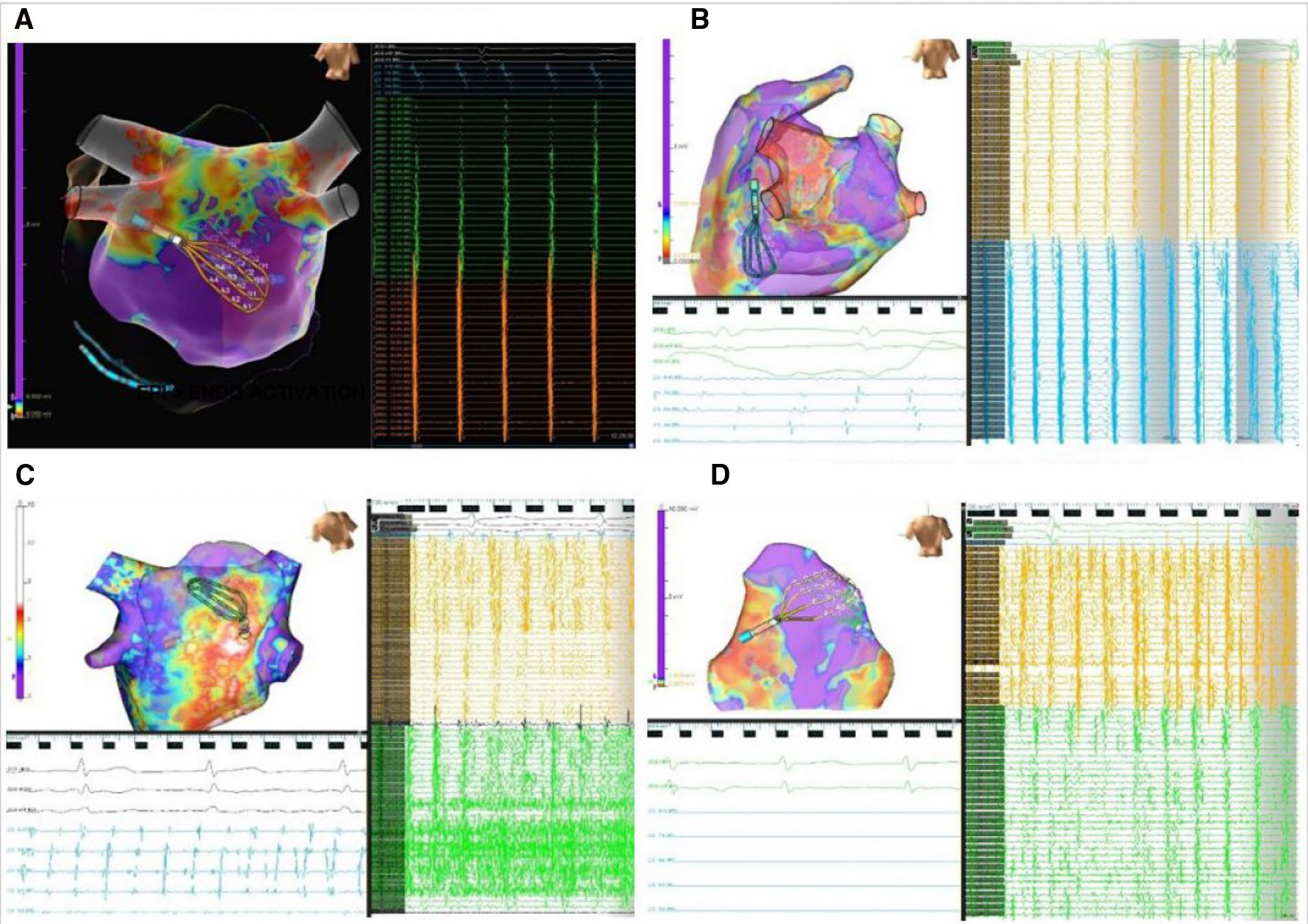
**Asynchronous epicardial and endocardial conduction. A**, Epicardial (Epi) leading endocardial (Endo) conduction. **B**, Endo leading Epi conduction. **C**, Greater Epi than Endo activation. **D**, Greater Epi>Endo activation. Representative episodes of Endo-Epi conduction during simultaneous Epi and Endo mapping. The figure shows 3-dimensional geometries, multipolar catheter (Advisor HD Grid, Abbott) positions on the Endo and Epi left atrial surfaces, and electrograms from these catheters and the decapolar catheter in the coronary sinus. **A**, Epi activity leading Endo activity. **B**, Endo activity leading Epi activity. **C** and **D**, Greater activation frequency on the Epi compared with the Endo surface.

Simultaneous endocardial and epicardial LAPW mapping was undertaken using 2 separate Grid catheters in 6 patients with AF. The remaining 10 patients in AF had sequential endocardial-epicardial mapping. During organized AF, one-to-one conduction was frequently observed with either epicardial-leading-endocardial activity or endocardial-leading-epicardial activity (Figure 6A and 6B). However, endocardial-epicardial asynchrony was universally seen with periods of less organized AF. In particular, episodes of more rapid epicardial compared with endocardial LAPW atrial activation during AF were observed in all 6 patients with simultaneous endocardial-epicardial mapping recordings (Figure 6C and 6D). In contrast, episodes of more rapid endocardial compared with epicardial LAPW AF activation were only seen in 2 patients with simultaneous endocardial-epicardial mapping recordings. In the remaining 10 patients in AF who had sequential endocardial then epicardial mapping, endocardial-epicardial asynchrony was also observed with episodes of more rapid epicardial compared with endocardial LAPW atrial activation during AF, as seen in patients who had simultaneous endocardial-epicardial mapping. The frequency of epicardial activity was significantly greater than endocardial activity at the mid-posterior wall (4.73±2.36 versus 2.40±1.42; *P*<0.001), mid left-inferior atrium (3.90±2.56 versus 1.60±1.43; *P*=0.005), and inferolateral left atrium (3.60±2.31 versus 1.90±1.79; *P*=0.03). There were 2 cases of more organized atrial activity suggestive of atrial flutter in which the epicardial and endocardial activity were similar, with 1:1 correspondence of LAPW activity. Patients in sinus rhythm had similar characteristics and 1:1 corresponding LAPW activity with no difference in endocardial and epicardial frequency (*P*>0.05 for all sites). Although the frequency of epicardial activity was greater during AF compared with sinus rhythm (*P*<0.05 for all sites), there was no significant difference in the frequency of endocardial activity during AF compared with sinus rhythm (*P*>0.05 for all sites).

### Endocardial and Epicardial Conduction Block

**Figure 7.**
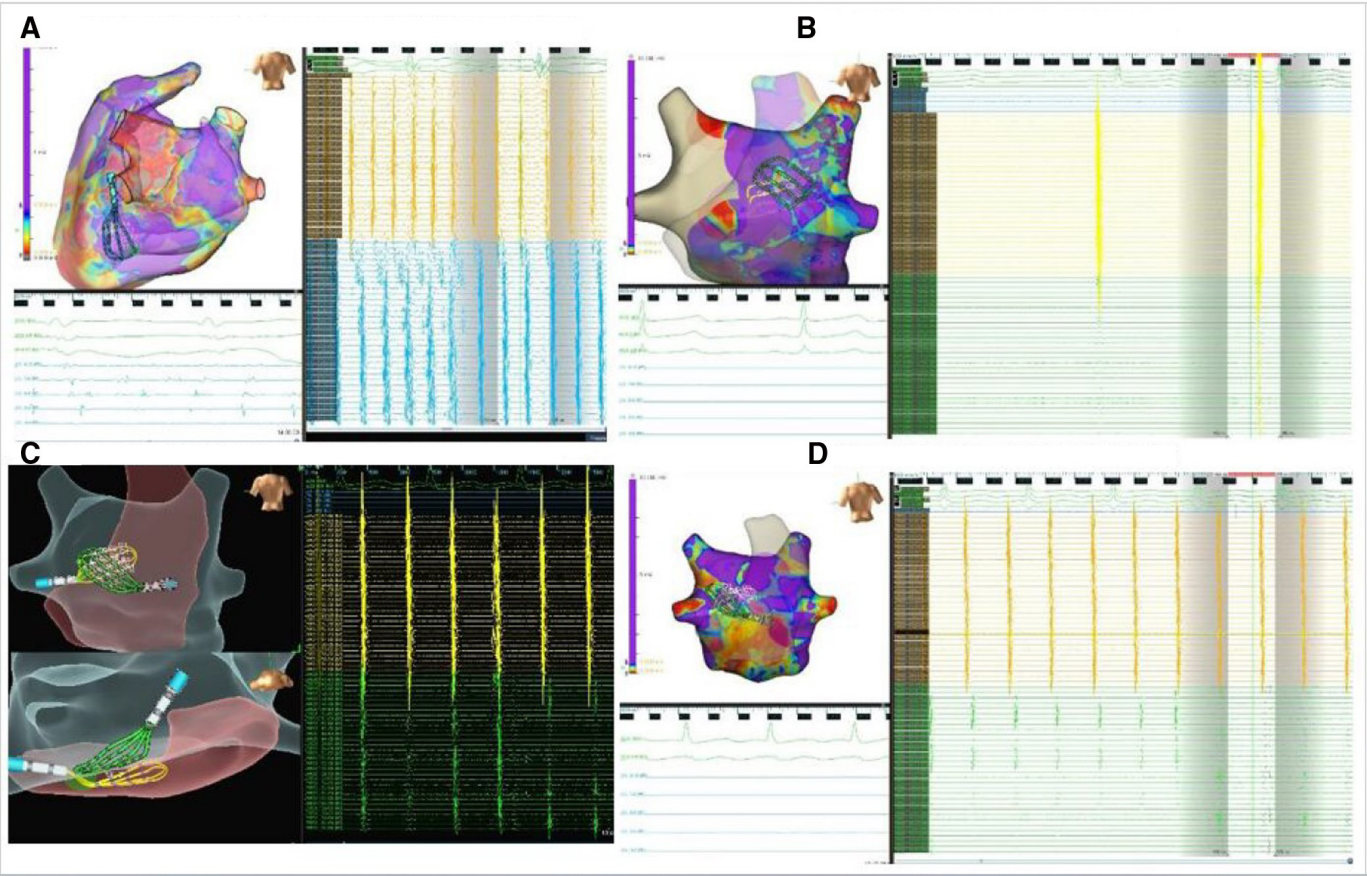
**Variable conduction block between endocardium and epicardium. A**, Endocardial (Endo) to epicardial (Epi) Wenckebach block during atrial fibrillation (AF). **B**, Epi to Endo Block during sinus rhythm (SR). **C**, Epi to Endo Block and Delay during AF. **D**, Epi to Endo Block During AF. For example, episodes of conduction block between the Endo and Epi left atrial posterior wall (LAPW) surfaces during simultaneous Epi and Endo mapping (SEEM). **A**, Single nonconducted beat due to Wenckebach conduction from the Endo to Epi surface during AF. **B**, Nonconducted beats from the Epi to Endo surface during sinus rhythm. **C**, Intermittent Epi to Endo block, and Epi to Endo delay, during AF. **D**, Regionally variable Epi to Endo block during AF.

**Figure 8.**
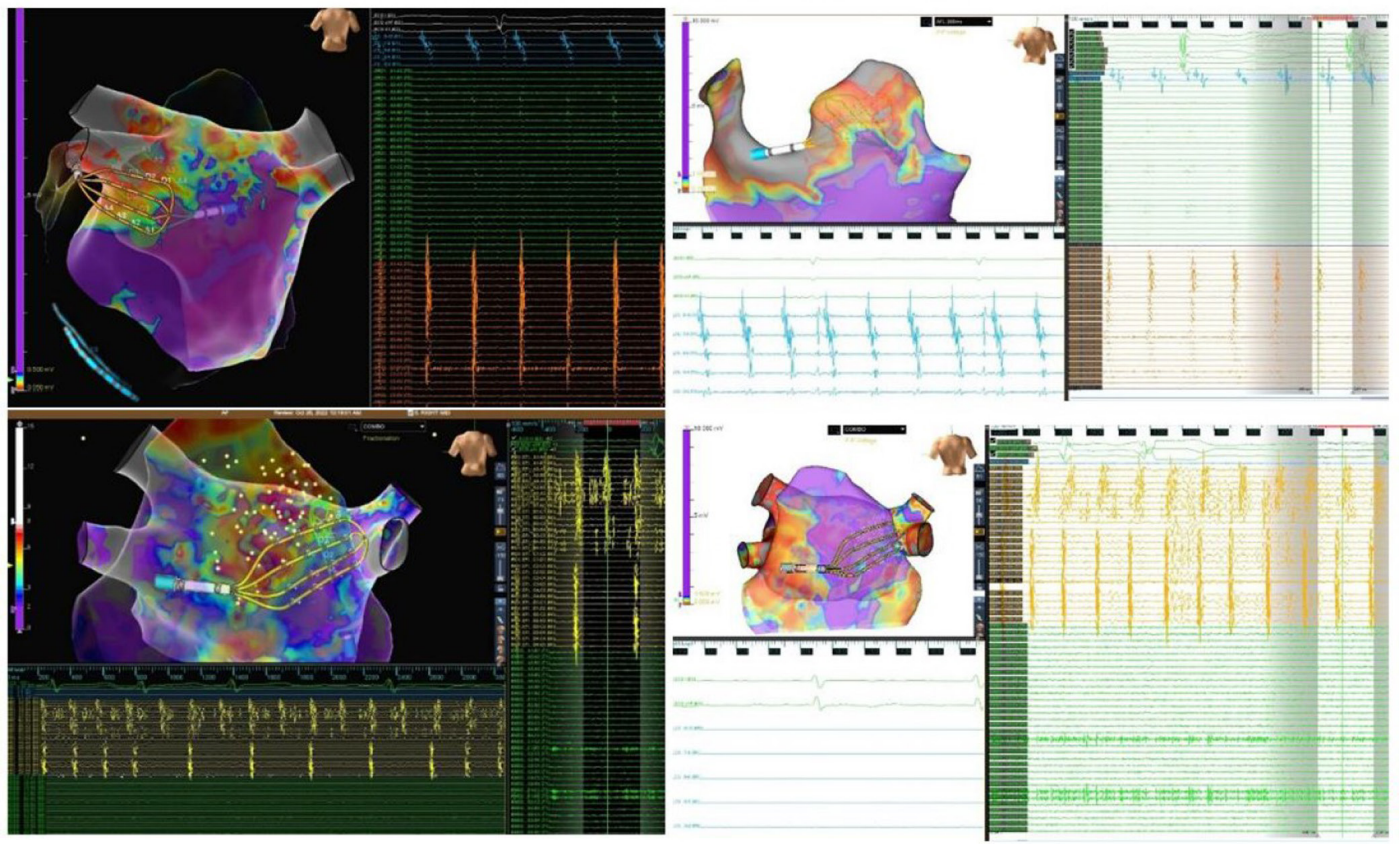
**Sustained epicardial-to-endocardial conduction block.** Representative episodes of sustained epicardial-to-endocardial conduction block during simultaneous epicardial and endocardial mapping (SEEM). The **bottom left** shows not only sustained epicardial-to-endocardial block but also transient 2:1 block on the epicardial surface. The figure shows 3-dimensional geometries, multipolar catheter (Advisor HD Grid, Abbott) positions on the endocardial and epicardial left atrial surfaces, and electrograms from these catheters and the decapolar catheter in the coronary sinus.

Functional conduction and conduction block were frequently observed between the endocardial and epicardial LAPW surfaces. Instances of isolated nonconducted beats, multiple blocked beats, and Wenckebach conduction were demonstrated during organized AF (Figure 7). Sustained conduction block to the endocardial LAPW with ongoing AF on the epicardial LAPW was also observed (Figure 8). Although simultaneous endocardial-epicardial mapping recordings during sinus rhythm were less frequent, epicardial-to-endocardial block was also observed (Figure 7).

### Clinical Follow-Up

Twenty-seven patients underwent a staged epicardial ablation followed by an endocardial ablation. All patients had LAA ligation during the epicardial ablation except for 1 patient due to an extremely rotated heart, and all were followed for adverse events and arrhythmias. There were no adverse events during the initial 7 days postepicardial ablation and LAA ligation. There was one delayed inflammatory pericardial effusion on day 14, not associated with hemodynamic compromise but was managed with pericardiocentesis. There were no 30-day adverse events associated with the follow-up endocardial ablation procedure. One patient died within 12 months due to sepsis and liver failure, which was not procedurally related. One patient was lost to follow-up after 6 months, resulting in 26 patients available to assess for freedom from arrhythmias at 6 months and 25 patients at 12 months. Freedom from atrial arrhythmias was 73% (19/26 patients) at 6 months, and 68% (17/25 patients) at 12 months. There were no embolic events during the 12 months of follow-up. Sixteen patients remained on oral anticoagulation therapy.

## Discussion

The present study represents the largest human series to characterize the dynamic nature of epicardial-endocardial asynchronous conduction of the LAPW during AF. Endocardial-epicardial LAPW asynchronous conduction was frequently observed in our cohort of persistent and long-standing persistent AF patients. During AF, there were greater epicardial compared with endocardial LAPW bipolar voltages, frequent epicardial-to-endocardial LAPW activation gradients, and prevalent conduction block between the epicardial and endocardial LAPW surfaces. Although less frequent, conduction block during sinus rhythm was also seen between the epicardial and endocardial LAPW surfaces. In areas of low endocardial bipolar voltage, endocardial unipolar data corresponded with normal epicardial bipolar voltages. These observations suggest a dynamic 3-dimensional arrhythmogenicity of the LAPW and the potential importance of the epicardial LAPW layer, with implications for the maintenance of persistent AF.

The asynchronous epicardial-endocardial nature of the LAPW has potential implications for endocardial mapping and ablation therapies. The observation that epicardial-to-endocardial conduction block can occur for prolonged periods of time could lead to the false interpretation of LAPW isolation when only endocardial mapping is performed. Even if such incomplete LAPW isolation is recognized, the dynamic nature of conduction block may render mapping for any epicardial-to-endocardial connections and detection of areas of functional epicardial breakthrough difficult. Endocardial LAPW isolation with radiofrequency catheter ablation is a common strategy utilized in patients with persistent and long-standing persistent AF, especially if there is a demonstration of durable PVI. However, the long-term efficacy of endocardial ablation in advanced nonparoxysmal AF is poor and is in part speculated to be related to the inability to produce durable LAPW isolation.^5^ Epicardial-to-endocardial conduction has been reported to be a factor in the recurrence of LAPW reconnection.^26^ Hybrid epicardial-endocardial ablation approaches have been demonstrated to be superior to endocardial ablation alone, implying that there is a lack of consistent creation of durable transmural lesions with endocardial ablation.^27–29^ Pulsed field ablation may help to prevent recurrence by producing a more homogenized ablation of the LAPW. However, initial results from the Manifest-AF registry did not demonstrate any difference between PVI and PVI plus pulsed field ablation of the LAPW.^30^

The limitations of contemporary endocardial ablation in persistent and long-standing persistent AF and the observations from the present study support the necessity of creating transmural lesions in the LAPW for increased freedom from atrial arrhythmias, especially in patients with enlarged left atrium and advanced nonparoxysmal AF.^28,29^ Residual endocardial unipolar LAPW voltage has been associated with arrhythmia recurrence after endocardial radiofrequency ablation of the LAPW.^31^ In the present study, the use of simultaneous endocardial-epicardial mapping demonstrated that in areas of no endocardial LAPW electrical potentials, endocardial unipolar mapping detected viable substrate that was not detected with endocardial bipolar mapping. These areas of normal endocardial unipolar voltage corresponded to normal epicardial bipolar voltage with robust electrical potentials, suggesting that endocardial unipolar mapping can detect far-field epicardial LAPW electrical potentials. Further studies are required to determine the utility of endocardial unipolar voltage mapping in detecting and targeting viable epicardial LAPW arrhythmic substrate.

Hybrid techniques involving both epicardial surgical and endocardial catheter ablation may be more likely to achieve durable transmural ablation of the LAPW compared with an endocardial-only approach.^32^ Concomitant LAA epicardial exclusion is also performed to potentially reduce thromboembolic risk and AF triggers.^32^ Although there is limited randomized trial data comparing hybrid epicardial-endocardial ablation therapy to catheter-only ablation, available data suggest hybrid approaches may be superior and imply that they might be more successful in creating permanent transmural lesions, especially in advanced forms of nonparoxysmal AF with enlarged left atrial size.^27,28,31^ For example, initial data on the subxiphoid hybrid epicardial-endocardial ablation approach with LAA ligation for nonparoxysmal AF described a 12-month freedom from atrial arrhythmias of 83%,^25^ significantly greater than reported efficacy rates of 43% with single-procedure endocardial ablation for persistent or long-standing persistent AF.^5^ In the subsequent randomized CONVERGE trial, the hybrid ablation group had a significantly greater 12-month freedom from atrial arrhythmias (67.7%) compared with the endocardial catheter ablation group (67.7% versus 50.0%, respectively).^27^ In the randomized HARTCAP-AF trial, 12-month freedom from atrial arrhythmias was also superior with hybrid ablation (89% versus 41%). These randomized results are consistent with previous observational data from experienced centers (70.7% versus 49.9% for hybrid ablation and endocardial ablation, respectively).^33^ Although not the focus of this study, our freedom from atrial arrhythmias at 12 months demonstrates similar efficacy rates as the previously published studies. It is also possible that patients with more advanced nonparoxysmal AF and greater left atrial remodeling may derive the greatest benefit from hybrid approaches. For example, while the addition of LAA ligation to PVI in the aMAZE did not improve overall 12-month atrial arrhythmia freedom, exploratory analyses suggested that LAA ligation might improve rhythm control in those with larger left atrial volumes, presumably by decreasing critical myocardial mass available for reentry.^34,35^ However, further prospective randomized trials are required to confirm the safety and efficacy of hybrid approaches versus catheter-based techniques, including the use of pulse field ablation technology, and to compare different hybrid components and lesion sets.

In addition to the clinical implications for mapping and ablation discussed above, our data begin to provide insight into AF mechanisms. Specifically, the demonstration of not only the presence of epicardial-endocardial asynchronous conduction, but also the dynamic nature of this with variable conduction patterns/block and voltage seen, supports a functional component and potential for reentry in 3 dimensions. Although epicardial-endocardial asynchronous conduction during AF has been studied in animal models^36,37^ and intraoperative right atrial patients,^38–40^ only a few isolated cases have been reported in the human left atrium.^41,42^ The present study thus represents the first large series to characterize epicardial-endocardial asynchrony in the human left atrium. Our data support the concept of multiple, often dissociated layers within remodeled atrial myocardium that can stabilize and perpetuate the fibrillatory process even when several of these layers are eliminated, for example, by nontransmural endocardial-only ablation. Indeed, there is significant evidence from prior ex vivo studies that the 3-dimensional architecture of atrial tissue facilitates epicardial-endocardial asynchrony, particularly in the setting of disease-related remodeling.^43,44^ Several important structural factors influencing epicardial-endocardial conduction include wall thickness, fiber angulation, and interstitial fibrosis that can lead to complex conduction dynamics, including intramural reentry.^43,44^ Our findings complement these prior experimental results, which together support the possibility that the 3-dimensional structure of the atrial wall may be critically important for the maintenance of AF.

### Limitations

Due to the persistent nature of many patients and logistical constraints, endocardial-epicardial mapping could not able to be undertaken in all patients during different rhythms (eg, AF and sinus rhythm). Had this been undertaken systematically, this may have provided further insight into the relative contribution of anatomic versus functional phenomena to the endocardial-epicardial asynchrony we observed. As most patients had persistent AF, there were limited opportunities to undertake systematic pacing evaluation to further characterize and confirm conduction phenomena observed; thus, our descriptive observations suggest, but do not prove, the 3-dimensional arrhythmogenicity of the LAPW. Furthermore, only a subset of patients underwent simultaneous endocardial-epicardial mapping, though findings from sequential mapping were consistent. In addition, our cohort had a predominance of long-standing persistent AF patients with enlarged left atrial size; the presence and complexity of endocardial-epicardial asynchrony, and relevance of this to ablation strategies, in earlier forms of persistent or paroxysmal forms of AF, requires further study. Several patients were on antiarrhythmic medications such as amiodarone and dofetilide, which may be a cause or contributor to our observations. However, these are frequently used medications, and thus our findings remain generalizable to AF patients in clinical practice. Our findings were observational in nature, and we did not seek to prospectively assess the arrhythmogenicity of the endocardial-epicardial asynchrony seen. Furthermore, as most patients had persistent AF, there was limited opportunity to undertake systematic pacing evaluation (by varying location, direction, rate, and other parameters) to further characterize and confirm conduction phenomena observed. However, our findings are suggestive of this possibility and provide the rationale for future studies evaluating specific endocardial-epicardial mechanisms or ablation strategies. Due to practical constraints in the setting of a clinical study, we focused on the LAPW given prior literature supporting the importance of this region for arrhythmogenicity, and did not characterize other atrial sites. Quantitative analyses were also not adjusted for multiple comparisons, given the exploratory nature of this work, and should thus be interpreted as hypothesis-generating, requiring confirmation in future studies. Although catheter positioning and contact were verified in several ways, and catheter distances comparable if not superior to prior clinical studies, the exact orientation could not be perfectly matched during all instances of simultaneous endocardial-epicardial mapping. Finally, given this was a clinical study undertaken during a minimally invasive procedure, assessment and correlation of atrial wall microstructure characteristics was not undertaken.

### Conclusions

Endocardial-epicardial LAPW asynchrony can be observed during human persistent AF. The variable conduction block commonly seen between the epicardial and endocardial surfaces during both AF and sinus rhythm supports the possible dynamic 3-dimensional arrhythmogenicity of the LAPW and the potential importance of the epicardial LAPW layer, with implications for mapping and ablation therapies. Future prospective studies are required to determine the significance of these findings to clinical ablation outcomes.

## Article Information

### Disclosures

Dr Wong reports that his institutions have received on his behalf research, lecture, or travel funding from Abbott Medical, Avant, Bayer, Boehringer Ingelheim, Boston Scientific, Medtronic, Microport, Novartis, Servier, St Jude Medical, and Vifor Pharma. Dr Lee is a part time medical director at AtriCure Inc. The other authors report no conflicts.
